# Metabolic pathways fueling the suppressive activity of myeloid-derived suppressor cells

**DOI:** 10.3389/fimmu.2024.1461455

**Published:** 2024-10-29

**Authors:** Oliver Goldmann, Eva Medina

**Affiliations:** Infection Immunology Research Group, Helmholtz Centre for Infection Research, Braunschweig, Germany

**Keywords:** myeloid-derived suppressor cells, metabolism, immunosuppression, tumor, metabolic reprogramming, infection

## Abstract

Myeloid-derived suppressor cells (MDSC) are considered an aberrant population of immature myeloid cells that have attracted considerable attention in recent years due to their potent immunosuppressive activity. These cells are typically absent or present in very low numbers in healthy individuals but become abundant under pathological conditions such as chronic infection, chronic inflammation and cancer. The immunosuppressive activity of MDSC helps to control excessive immune responses that might otherwise lead to tissue damage. This same immunosuppressive activity can be detrimental, particularly in cancer and chronic infection. In the cancer setting, tumors can secrete factors that promote the expansion and recruitment of MDSC, thereby creating a local environment that favors tumor progression by inhibiting the effective immune responses against cancer cells. This has made MDSC a target of interest in cancer therapy, with researchers exploring strategies to inhibit their function or reduce their numbers to improve the efficacy of cancer immunotherapies. In the context of chronic infections, MDSC can lead to persistent infections by suppressing protective immune responses thereby preventing the clearance of pathogens. Therefore, targeting MDSC may provide a novel approach to improve pathogen clearance during chronic infections. Ongoing research on MDSC aims to elucidate the exact processes behind their expansion, recruitment, activation and suppressive mechanisms. In this context, it is becoming increasingly clear that the metabolism of MDSC is closely linked to their immunosuppressive function. For example, MDSC exhibit high rates of glycolysis, which not only provides energy but also generates metabolites that facilitate their immunosuppressive activity. In addition, fatty acid metabolic pathways, such as fatty acid oxidation (FAO), have been implicated in the regulation of MDSC suppressive activity. Furthermore, amino acid metabolism, particularly arginine metabolism mediated by enzymes such as arginase-1, plays a critical role in MDSC-mediated immunosuppression. In this review, we discuss the metabolic signature of MDSC and highlight the therapeutic implications of targeting MDSC metabolism as a novel approach to modulate their immunosuppressive functions.

## 1 Origin and phenotype of MDSC

Myeloid cells comprise a diverse array of cell types with specialized functions that play critical roles in the innate immune response to pathogens, maintaining tissue homeostasis, and orchestrating inflammatory responses (1). However, under pathological conditions such as cancer, autoimmunity, or chronic infection, immature myeloid cells expand rapidly, remain in an undifferentiated state, and acquire immunosuppressive properties (2, 3). Based on their phenotype and suppressive activity, this aberrant myeloid cell population has been termed myeloid-derived suppressor cells (MDSC) (4). MDSC have been classified into two major subsets based on their lineage: monocytic MDSC (M-MDSC) and granulocytic or polymorphonuclear MDSC (PMN-MDSC) (3, 5, 6). The M-MDSC subset shares phenotypic and functional similarities with monocytes, whereas the PMN-MDSC subset exhibits characteristics reminiscent of neutrophils. M-MDSC and G-MDSC subsets are commonly identified using the following phenotypic markers: HLA-DR^-/lo^CD33^+^CD11b^+^CD14^+^CD15^-^ (M-MDSC) and CD11b^+^CD15^+^HLA-DR^low^CD66b^+^ (G-MDSC) in humans and CD11b^+^Ly6G^−^Ly6C^hi^ (M-MDSC) and CD11b^+^Ly6G^+^Ly6C^lo^ (G-MDSC) in mice (3). However, within these broad categories, there is further diversity in terms of surface marker expression and gene expression profiles (7–9).

Due to their potent suppressive effect on T cell responses, MDSC represent a significant obstacle in both natural anti-tumor immunity and cancer immunotherapy. Thus, MDSC interfere with the ability of the immune system to mount an effective anti-tumor response (10–12). Immunotherapy has emerged as a revolutionary approach to cancer treatment (13, 14). Unlike traditional treatments such as chemotherapy or radiation therapy, which target cancer cells directly, immunotherapy harnesses the power of the immune system to fight cancer (13, 14). Due to their immunosuppressive activity, MDSC play an important role in dampening the efficacy of immunotherapy in cancer (15–18). Therefore, targeting MDSC or neutralizing their immunosuppressive effects represents a promising strategy to improve the efficacy of immunotherapy in cancer (15, 16).

There is a growing body of evidence highlighting the important role of MDSC in regulating immune responses to various pathogens (19, 20). Their potent ability to suppress effector immune cells can inhibit the efficacy of the immune system to effectively control infections (19, 20). This phenomenon is particularly relevant in the context of chronic infections, where prolonged MDSC-mediated immunosuppression can create a favorable environment for pathogen persistence and immune evasion (21, 22). Thus, inhibition of MDSC function may potentially enhance the efficacy of the immune system against infectious agents.

MDSC expansion and differentiation are influenced by a variety of factors, including tumor-derived factors such as granulocyte-macrophage colony-stimulating factor (GM-CSF) and granulocyte colony-stimulating factor (G-CSF), and the signaling pathways triggered by these molecules, such as STAT3 (23, 24). Inflammatory mediators, including IL-1*β*, IL-6, and S1008/9 have been shown to induce MDSC accumulation in tumors (25–29). Emergency and/or extramedullary myelopoiesis may also be an important source of MDSC during pathological conditions (6, 7). Myelopoiesis occurs under steady-state conditions within the bone marrow microenvironment, ensuring the production of sufficient neutrophils, monocytes, and other myeloid cells to maintain immune surveillance (30). However, acute insults during pathological conditions such as cancer or infection can trigger emergency myelopoiesis, a rapid response mechanism aimed at replenishing myeloid cells to combat the insult (31). In some cases, when the capacity of the bone marrow is overwhelmed, extramedullary myelopoiesis occurs, where hematopoietic activity takes place outside the bone marrow, typically in the liver and spleen (32). Both emergency and extramedullary hematopoiesis have been shown to contribute to the expansion of MDSC observed during chronic *Staphylococcus aureus* infection in mice (7), as well as in other pathological conditions such as sepsis (33).

The reason why MDSC are arrested at an immature stage and do not continue to mature is still a matter of debate. However, it appears that metabolism may play a key role in the arrest of maturation. In this regard, we have reported that an important factor limiting MDSC maturation in infected tissue is nutrient limitation in the microenvironment (7). Immature myeloid cells require a significant amount of energy to mature into functional immune cells. This high energy demand is necessary to support various cellular processes such as cell division, protein synthesis, and the development of specialized functions such as phagocytosis and cytokines production (34). High consumption of nutrients such as glucose can lead to its rapid depletion in the cellular microenvironment. In this regard, we have recently reported an association between high glucose consumption and disruption of MDSC maturation processes (7).

## 2 Glucose metabolism fuels the suppressive activity of MDSC

Every cell requires a constant supply of ATP and the synthesis of macromolecules for essential cellular activities. To meet these energy demands, cells rely on a network of interconnected pathways, including glycolysis, the tricarboxylic acid (TCA) cycle, and oxidative phosphorylation (OXPHOS). Essentially, these metabolic pathways form the backbone of cellular energy production and molecular synthesis, ensuring the cell survival and functionality. In the case of myeloid cells, different subsets exhibit distinct metabolic preferences to support their diverse functions in the immune system (35). For example, M1 macrophages, also known as classically activated macrophages, typically rely on glycolysis as their primary metabolic pathway for energy generation and exhibit low levels of OXPHOS and fatty acid oxidation (FAO) (36). This preference for glycolysis is associated with their proinflammatory functions, such as phagocytosis and the production of proinflammatory cytokines such as interleukin-1β (IL-1β), tumor necrosis factor-alpha (TNF-α), and interleukin-6 (IL-6). In contrast, M2 macrophages, also known as alternatively activated macrophages, are more dependent on FAO and OXPHOS for energy production (36, 37). This metabolic preference is associated with their anti-inflammatory and tissue repair functions, as well as their role in resolving inflammation and promoting tissue homeostasis (36). Neutrophils utilize a variety of metabolic pathways including the TCA, OXPHOS, FAO, and pentose phosphate pathway (PPP), to meet their energetic and functional requirements (38, 39). These pathways provide the necessary energy, biosynthetic precursors, and production of reactive oxygen species (ROS) essential for neutrophil activities such as phagocytosis, respiratory burst, and chemotaxis (40).

MDSC have different metabolic profiles than mature myeloid cells. In general, MDSC rely on glycolysis to meet their energy needs. In this regard, the metabolic activity of MDSC has been reported to be similar to that of cancer cells. Cancer cells rely on glycolysis rather than OXPHOS for energy production, even in the presence of oxygen, a phenomenon known as the “Warburg effect” (41, 42). This metabolic adaptation allows cancer cells to generate ATP and metabolic intermediates necessary for cell growth and proliferation, while also providing building blocks for biosynthesis (41, 42). In the presence of oxygen, glucose is metabolized by enzymatic reactions in the cell cytoplasm to pyruvate. Pyruvate is then transported to the mitochondria and enters the TCA cycle, where it is oxidized to produce NADH and ATP. However, in aerobic glycolysis, a significant proportion of the pyruvate that is not transported to the mitochondria to enter the TCA cycle is converted to lactate in the cytosol by the enzyme lactate dehydrogenase (LDH) (43). The advantages of aerobic glycolysis include the rapid generation of ATP and the production of metabolic intermediates (43). In addition, lactate produced by cancer cells induces acidification of the tumor microenvironment and contributes to the ability of tumors to evade the immune system and promote tumor progression (44).

**Figure 1:**
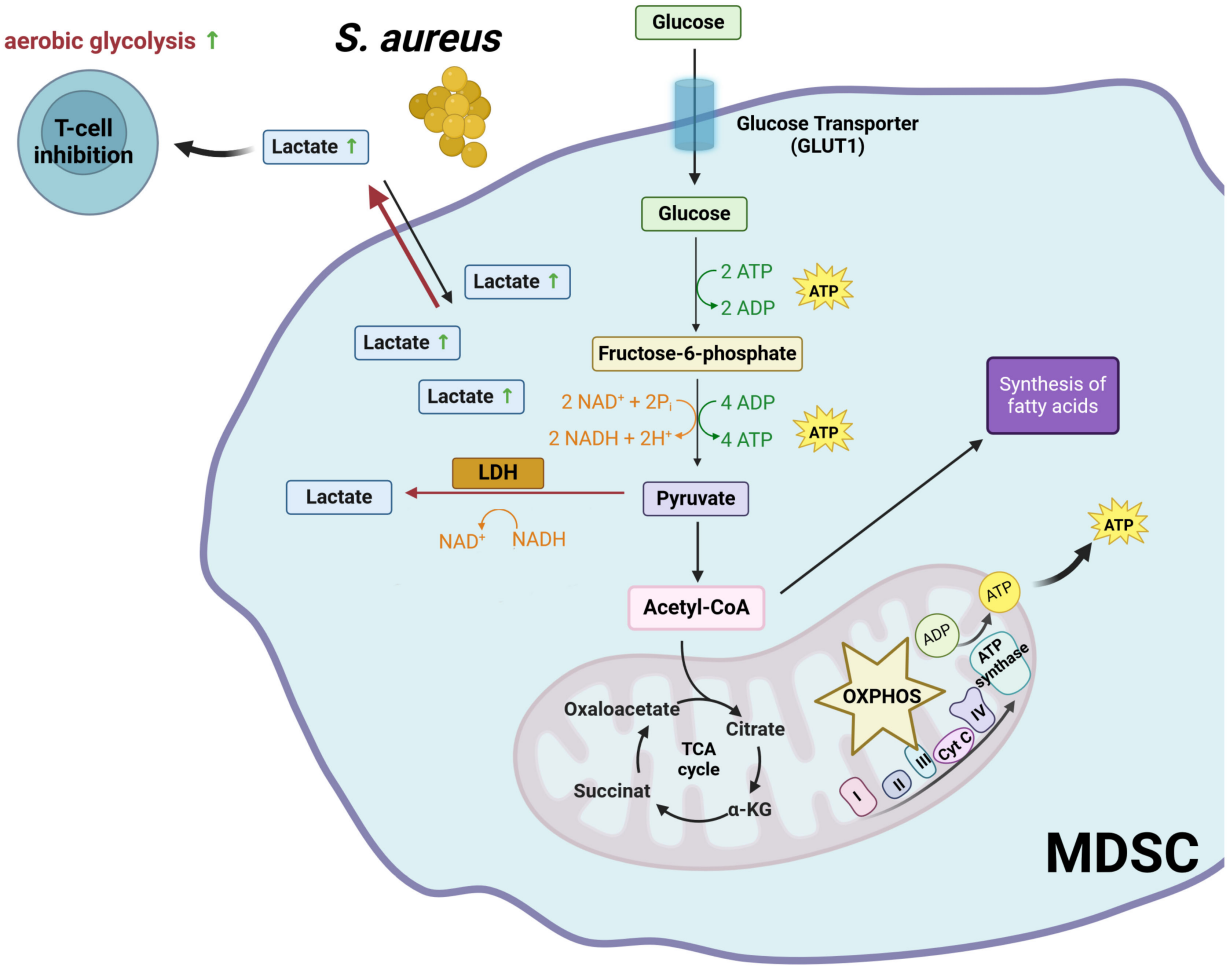
Glycolysis-mediated immunosuppressive mechanisms of MDSC. LDH, lactate dehydrogenase; NAD+, oxidized nicotinamide adenine dinucleotide; NADH, reduced nicotinamide adenine dinucleotide; OXPHOS, oxidative phosphorylation; TCA,tricarboxylic acid cycle. Created with BioRender.com.

Like cancer cells, MDSC rely heavily on aerobic glycolysis to meet their energy needs and support their rapid proliferation (7, 45). MDSC also induce an immunosuppressive environment that inhibits protective immune responses against tumor cells in the context of cancer (45) and against pathogens in the context of infection (7). Recently, our group has reported that MDSC generated during chronic *S. aureus* infection can undergo aerobic glycolysis, leading to the production of high levels of lactate (7). This creates a lactate-rich microenvironment that can inhibit the function of nearby immune cells, including CD4+ T cells, which play a critical role in orchestrating the immune response against *S. aureus* (46). Upon activation, CD4+ T cells must undergo specific metabolic reprogramming to meet the energy demands associated with proliferation and effector functions. Activated CD4+ T cells switch their metabolism from OXPHOS to aerobic glycolysis, similar to what cancer cells and MDSC do. This metabolic switch allows CD4+ T cells to rapidly generate ATP and biosynthetic intermediates required for cell proliferation, cytokine production, and other effector functions (47–50). The lactate molecules generated during aerobic glycolysis must be exported from activated CD4+ T cells to maintain the redox balance and ensure the continuation of glycolysis. Monocarboxylate transporters (MCTs) are responsible for the export of lactate across the plasma membrane. These transporter proteins facilitate the movement of lactate together with protons (H^+^) across the membrane, driven by their concentration gradients (51, 52). High concentrations of extracellular lactate can interfere with CD4+ T cell activation by reversing the lactate flux. We have provided evidence demonstrating that MDSC, which accumulate in high numbers during chronic *S. aureus* infection, release elevated levels of lactate into the tissue microenvironment, leading to the accumulation of intracellular lactate in activated CD4+ T cells (46). When the intracellular concentration of lactate in activated CD4+ T cells is high, the lactate dehydrogenase catalyzes the reversible conversion of lactate back to pyruvate. This process results in a redox shift from NAD+ to NADH (53). As NAD+ is essential for maintaining the activity of several enzymes involved in glycolysis, low levels of NAD+ lead to a slowing or stoppage of the glycolytic process (53). This results in inhibition of CD4+ T cell activation, proliferation, and cytokine production, thereby suppressing their ability to control *S. aureus* (46) (**Figure 1**).

## 3 Hypoxic conditions affect MDSC metabolism

The metabolic phenotype of MDSC can change depending on factors such as oxygen levels, nutrient availability, and the presence of inflammatory mediators. Indeed, metabolic plasticity stands out as a crucial aspect of MDSC. For example, MDSC often encounter hypoxia (low oxygen levels) in tumor microenvironment and must adapt their metabolic activity to these hypoxic conditions (54–56). The main molecular mechanism underlying the cellular response to hypoxia is mediated by the transcription factor hypoxia-inducible factor 1 (HIF-1) (57, 58). HIF-1 controls the expression of a large number of genes, including several involved in glucose transport and metabolism (59). HIF-1 is a heterodimeric transcription factor consisting of two subunits: HIF-1α and HIF-1β (60). HIF-1β is constitutively expressed, whereas the stability and activity of HIF-1α is regulated by oxygen levels (60). Under normoxic conditions (normal oxygen levels), HIF-1α is hydroxylated by prolyl hydroxylase enzymes, leading to its degradation via the ubiquitin-proteasome pathway (61). However, under hypoxic conditions, HIF-1α is stabilized and translocates to the nucleus, where it forms a complex with HIF-1β (62). This HIF-1 complex then binds to hypoxia response elements in the promoter regions of target genes, activating their transcription and initiating various adaptive responses to hypoxia (63). In the hypoxic tumor environment, HIF-1α has been shown to induce upregulation of the genes encoding arginase 1 and nitric oxide synthase with concomitant downregulation of the gene encoding NADPH oxidase 2 (64). These changes induced by HIF-1α in MDSC enhanced their ability to suppress T cell functions in the tumor environment (64).

It has also been reported that MDSC are able to display phenotypic and functional characteristics of both classically activated (M1-like) and alternatively activated (M2-like) macrophages in tumor-bearing mice (65). Sirtuin 1 (SIRT1), also known as NAD-dependent deacetylase sirtuin-1, appears to play a pivotal role in dictating the fate of MDSC as they differentiate into M1 or M2 phenotypes (66). SIRT1 plays a critical role in cellular metabolism and stress response by acting as a metabolic sensor (67). It regulates gene expression and cellular processes through its deacetylase activity, which can affect chromatin structure and protein function (67). This ability allows SIRT1 to modulate several physiological functions, including energy metabolism and stress response (67). SIRT1 regulates MDSC differentiation into M1 or M2 phenotypes by targeting the HIF-1α pathway, thereby inducing glycolytic reprogramming (66). Glucocorticoid receptor activation has also been shown to promote MDSC suppressive activity by inhibiting HIF-1α, thereby disrupting MDSC glycolytic activity and metabolism (68).

HIF-1α has also been shown to influence MDSC during *Leishmania donovani* infection (69). In this context, HIF-1α plays an important role in promoting the establishment of chronic infection (69). Monocytes differentiate into MDSC in the chronically inflamed spleen of mice infected with *L. donovani.* HIF-1α activation contributes to the immunosuppressive environment of the inflamed spleen by enhancing the functions of MDSC while dampening the ability of Th1 cells to control Leishmania infection (69).

**Figure 2:**
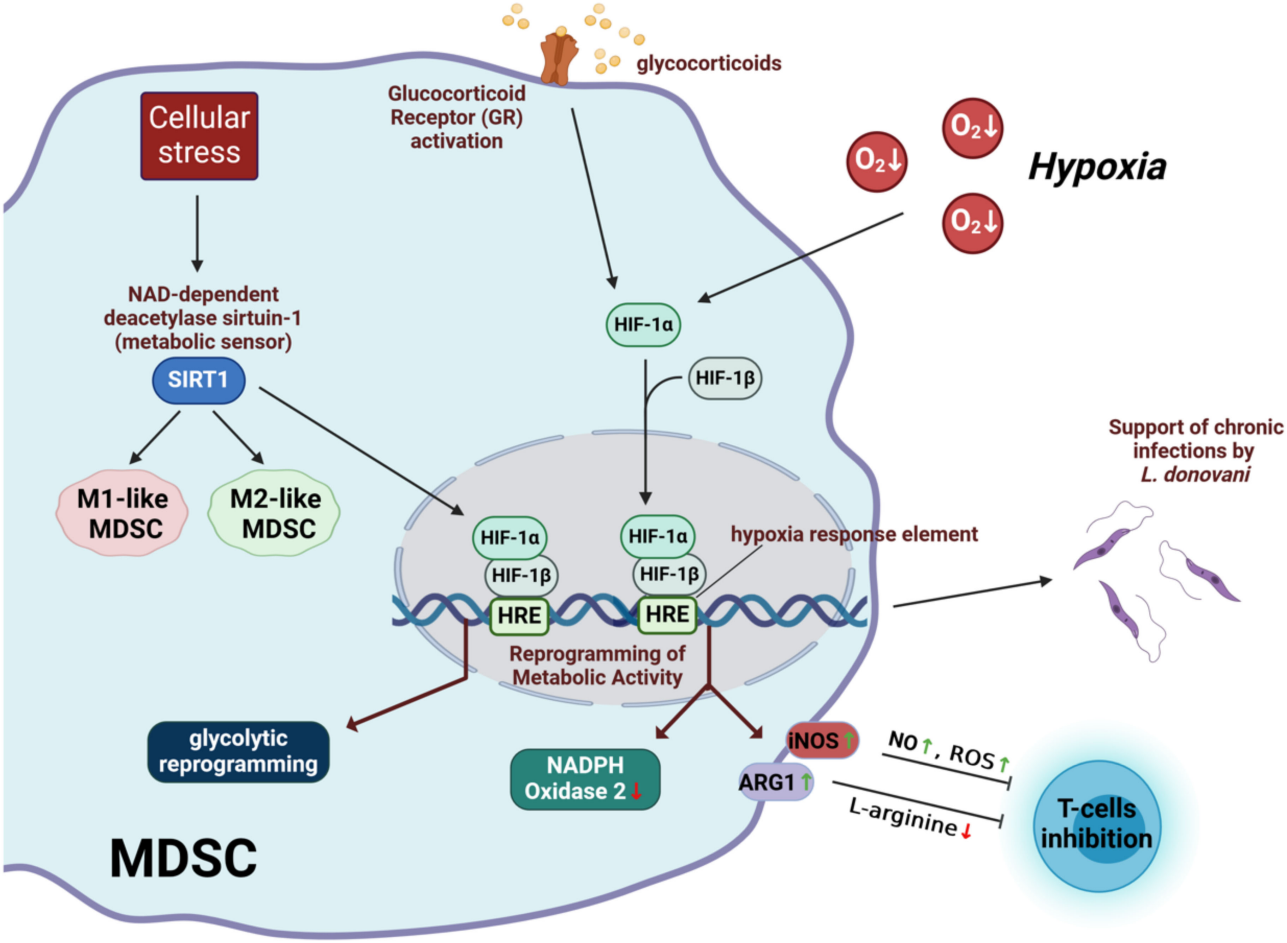
Immunosuppressive mechanisms of MDSC affected by hypoxia. HIF-1, hypoxia-inducible factor 1; SIRT1, irtuin 1; iNOS, inducible nitric oxide synthase; ARG1, arginase 1; NO, nitric oxide; ROS, reactive oxygen radicals; HRE, hypoxia-responding elements. Created with BioRender.com.

The mechanisms underlying the influence of hypoxia on MDSC-mediated immunosuppression are summarized in **Figure 2**.

## 4 Interplay between lipid metabolism and MDSC function

While glycolysis is the main metabolic pathway used by MDSC to generate energy and precursors for biosynthetic pathways, they can also utilize other energy pathways such as FAO. This pathway, also known as beta-oxidation, is a metabolic process in which fatty acids are broken down to produce energy (70, 71). FAO mainly takes place in the mitochondria of cells (70, 71). During this process, fatty acids are first activated in the cytoplasm before they can be transported into the mitochondria (72). This activation involves the conversion of a fatty acid into fatty acyl-CoA by acyl-CoA synthetase (72). However, long-chain fatty acyl-CoA molecules cannot cross the mitochondrial membrane directly and must be transported via the carnitine shuttle (73). In this process, the fatty acyl group is transferred to carnitine by the enzyme carnitine palmitoyltransferase I located on the outer mitochondrial membrane, forming fatty acyl-carnitine (73). This compound is then transported across the inner mitochondrial membrane by a translocase enzyme (73). On the inner mitochondrial side, carnitine palmitoyltransferase II transfers the fatty acyl group back to CoA, forming fatty acyl-CoA, which undergoes beta-oxidation to produce ATP (73). MDSC have been reported to upregulate fatty acids uptake and increase FAO after tumor infiltration (74). This increase in lipid metabolism appears to be related to the immunosuppressive activity of MDSC, as inhibition of FAO reduces the inhibitory effect of MDSC on T cells and suppresses the production of inhibitory cytokines (74). Tumor-derived factors such as G-CSF and GM-CSF, together with the resulting signaling cascade, increase the expression of lipid transport receptors (75). This increased lipid uptake leads to enhanced oxidative metabolism and activation of ROS-mediated immunosuppressive mechanisms (75). Consequently, the immunosuppressive effect of MDSC could be abrogated by deleting lipid transporters or by inhibiting the underlying signaling pathways (75).

It has also been reported that activation of STAT5 by GM-CSF induces high levels of fatty acid transport protein 2 (FATP2) expression in human PMN-MDSC (76). FATP2 plays a critical role in lipid metabolism by facilitating the uptake of long-chain fatty acids, such as arachidonic acid, into the cells. Arachidonic acid is a polyunsaturated fatty acid that serves as an important precursor for the biosynthesis of several eicosanoids, including prostaglandins. Prostaglandin E_2_, in particular, has been implicated in the suppressive mechanisms of MDSC in tumors (27, 77). Upregulation of FATP2 in human PMN-MDSC results in increased uptake of arachidonic acid and production of prostaglandin E_2_. Pharmacological inhibition of FATP2 has been shown to abolish the suppressive effect of PMN-MDSC and delay tumor progression in mice (76). This supports the idea that targeting lipid metabolism in MDSC may enhance the efficacy of cancer immunotherapy.

**Figure 3:**
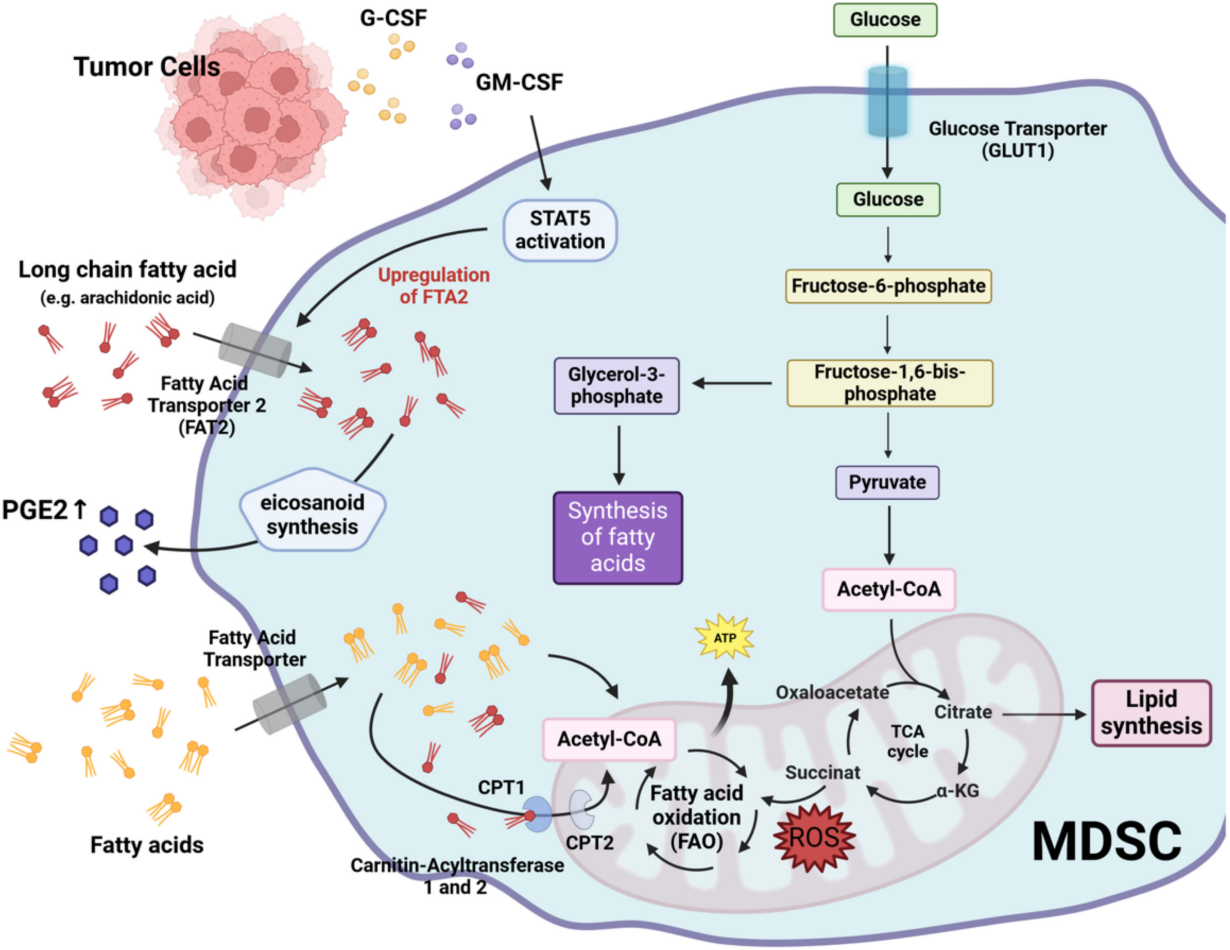
Lipid metabolism-mediated immunosuppressive mechanisms of MDSC. G-CSF, granulocyte colony-stimulating factor; GM-CSF, granulocyte-macrophage colony-stimulating factor; STAT5, signal transducer and activator of transcription 5; PGE2, prostaglandin E2; FTA2, fatty acid transport protein 2; CPT1, carnitine palmitoyltransferase I; CPT2, carnitine palmitoyltransferase II. Created with BioRender.com.

**Figure 3** outlines the mechanisms by which lipid metabolism contributes to MDSC-mediated immunosuppression.

## 5 MDSC function and amino acid metabolism

An important mechanism used by MDSC to suppress the activity of effector T cells is to deplete the microenvironment of amino acids that are essential for T cell functionality (e.g. arginine, glutamine and tryptophan). L-arginine is critical for T cell activation as it supports the synthesis of proteins and polyamines necessary for cell growth and proliferation (78, 79). MDSC express high levels of arginase 1 and nitric oxide synthase 2, which can deplete L-arginine in the tissue and thereby inhibit T cell responses (80). Arginase 1 catalyzes the conversion of arginine into ornithine and urea. Therefore, increased arginase 1 activity may lead to depletion of extracellular arginine. It has also been reported that L-arginine depletion can cause down-modulation of CD3ζ (CD3 zeta chain) (81). CD3ζ (CD3 zeta chain) is a critical component of the T cell receptor (TCR) complex and plays an essential role in TCR signal transduction. Down-modulation of CD3ζ can impair TCR signaling, thereby affecting T cell function and immune responses.

MDSC can also promote immunosuppression by upregulating indoleamine 2, 3-dioxygenase (IDO), which induces the expansion of regulatory T cells (82–84). IDO is a cytosolic enzyme that catalyzes the first and rate-limiting step in the breakdown of tryptophan along the kynurenine degradation pathway (85). IDO modulates T cell function through the catabolism of tryptophan, an essential amino acid that must be obtained from the diet (86). The degradation of tryptophan by IDO leads to local depletion of this amino acid in the microenvironment. T cells are particularly sensitive to tryptophan levels and reduced availability inhibits their proliferation and induces cell cycle arrest in the G1 phase (87). In addition, kynurenine metabolites generated during tryptophan catabolism can induce the expression of forkhead box P3 (FoxP3), which is critical for the differentiation and function of regulatory T cells (88, 89). Kynurenines also inhibit retinoic acid receptor-related orphan receptor-γt (RORγt), which promotes the differentiation of Th17 cells (90).

Glutaminolysis, a metabolic process in which the amino acid glutamine is broken down to produce energy and various metabolic intermediates, has been shown to play a role in the maturation and immunosuppressive activities of MDSC (91, 92). Thus, by converting some of the carbon derived from glutamine to lactate, MDSC are thought to contribute to the acidification of the tumor microenvironment (91). Targeting glutaminolysis has therefore been proposed as a way to improve the anti-tumor immune response (93). Indeed, inhibition of glutamine metabolism in MDSCs resulted in cell death and conversion to inflammatory macrophages (93). Furthermore, blocking glutamine metabolism also inhibited IDO expression, leading to a decrease in kynurenine levels and inhibition of metastasis (93).

**Figure 4:**
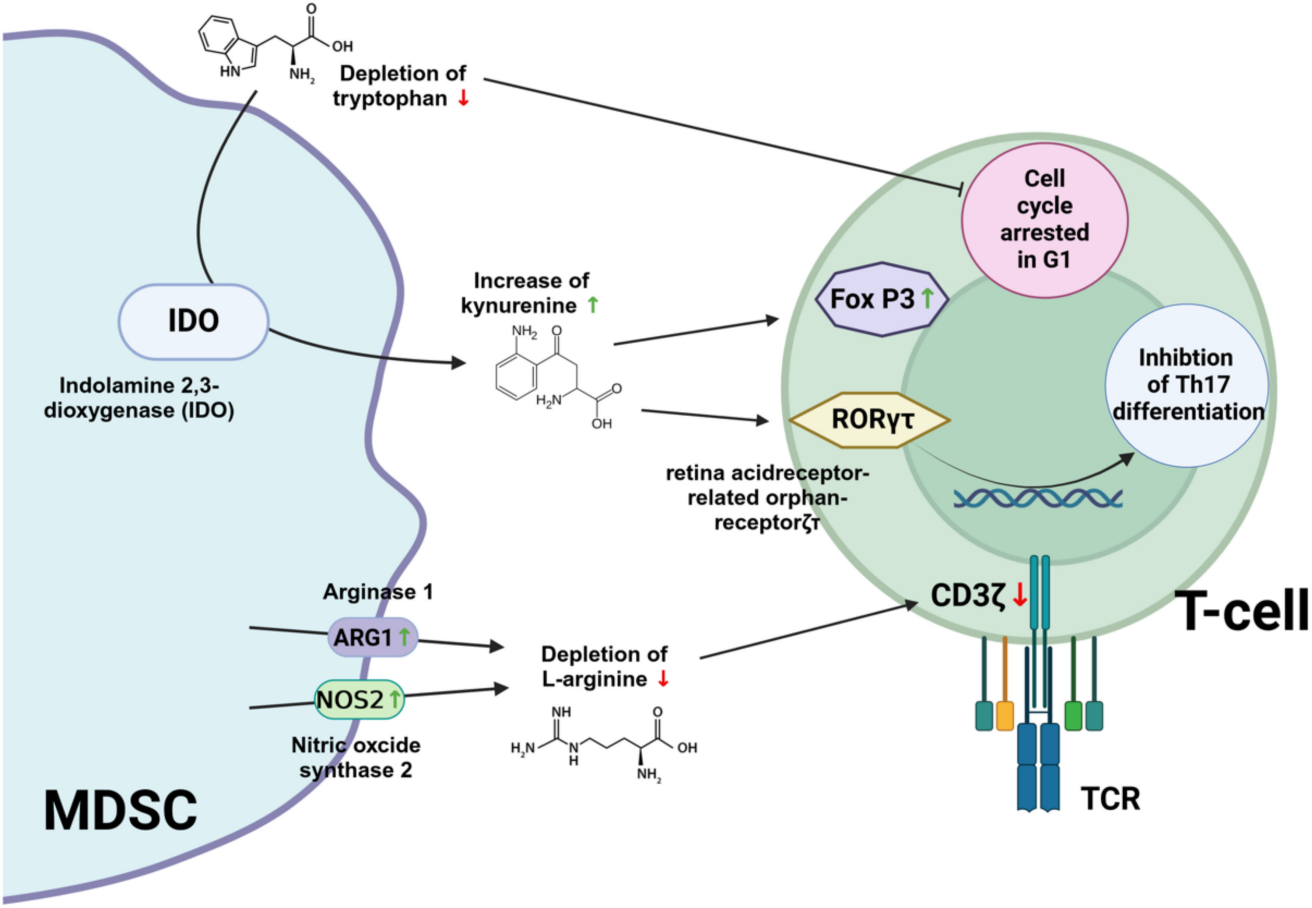
Immunosuppressive mechanisms of MDSC supported by amino acids metabolism. IDO, ndolamin-2,3-Dioxygenase; FOX P3, Forkhead box P3; RORγt, retinoic acid receptor-related orphan receptor-γt; TCR, T cell receptor; CD3ζ, CD3 zeta chain. Created with BioRender.com.

**Figure 4** illustrates how amino acid metabolism supports the immunosuppressive activities of MDSC.

## 6 Targeting MDSC metabolism to improve cancer immunotherapy

Immunotherapy has been introduced in recent years as a treatment for several types of cancer (14, 94). Several modalities of immunotherapy are currently being investigated, including monoclonal antibodies targeting tumor-associated antigens, cancer vaccines, adoptive cell-based immunotherapies, and non-specific enhancement of the immune system using interferons or toll-like receptor ligands (95). The aim of immunotherapy is to stimulate immune cells to eliminate tumor cells, rather than directly targeting the tumors themselves. However, many patients experience limited benefit from immunotherapy and only a small proportion of treated patients have shown improved survival (96). One of the reasons for treatment failure is the presence of immunosuppressive mechanisms in the tumor environment that dampen the effectiveness of the anti-tumor immune response. Therefore, a critical issue in improving the efficacy of immunotherapy in cancer is overcoming the immunosuppression in the tumor environment (14, 94). In this context, there is increasing evidence that MDSC play a critical role in promoting immunosuppression in several types of cancer (97, 98). As many of the immunosuppressive mechanisms of MDSC are mediated by their metabolic activity, combining immunotherapy with agents that target MDSC metabolism could be an effective means of enhancing the efficacy of cancer immunotherapy. Numerous studies have been conducted to evaluate the efficacy of these combined approaches (16). For example, the combination of IDO inhibitors and immune checkpoint inhibitors holds promise as an effective and well-tolerated approach to cancer therapy. As mentioned above, the IDO enzyme plays a role in immune suppression by catalyzing the breakdown of tryptophan to kynurenine, which in turn suppresses T cell function and promotes the development of regulatory T cells (82–84). Immune checkpoint inhibitors, such as anti-PD-1 (programmed cell death protein 1) and anti-CTLA-4 (cytotoxic T-lymphocyte-associated protein 4) antibodies, work by blocking the checkpoints that cancer cells use to evade immune detection (99). Therefore, combining IDO inhibitors with immune checkpoint inhibitors may enhance anti-tumor immunity because IDO inhibitors can prevent tryptophan depletion in the tumor microenvironment. This can restore the proliferation and function of T cells, making them more effective in targeting tumor cells. In preclinical studies, blocking both IDO1 and an immune checkpoint pathway showed more effective control of tumor growth than inhibiting the immune checkpoint alone (100, 101). A recent clinical trial demonstrated favorable outcomes and improved survival in patients with metastatic melanoma following treatment with a therapeutic peptide vaccine targeting IDO and PD-L1 in combination with nivolumab, a monoclonal antibody that acts as a checkpoint inhibitor by binding to PD-1 on the surface of effector T cells (102). An ongoing phase III trial is evaluating the IDO/PD-L1 vaccine in combination with the anti-PD-1 therapy pembrolizumab in patients with advanced melanoma (NCT05155254). Other clinical trials are evaluating the combination of IDO inhibitors, including epacadostat, navoximod and BMS-986205, with immune checkpoint inhibitors in cancer patients, with early results showing that these combination therapies are well tolerated and have some activity (103, 104). In addition, IDO inhibitors are being tested in combination with radiotherapy, chemotherapy and anti-tumor vaccines in clinical trials (105). Researchers are also investigating dual IDO-TDO inhibitors and novel inhibitors of the Trp-Kyn-AhR pathway, such as Kyn-degrading enzymes, direct AhR antagonists and tryptophan mimetics (106).

Combination therapy including an anti-CD40 agonist with celecoxib has been shown to reduce ARG1 expression in MDSC and improve survival in GL261 glioma bearing mice compared to monotherapy alone (107).

As mentioned in previous sections, targeting the fatty acid metabolism of MDSC has also been shown to inhibit their immunosuppressive activity (74). In this regard, etomoxir, a selective inhibitor of carnitine palmitoyltransferase 1, significantly delayed tumor growth in several murine tumor models in a T cell-dependent manner (74). The selective FATP2 inhibitor lipofermata, used alone or in combination with checkpoint inhibitors, inhibited PMN-MDSC activity and significantly delayed tumor progression in mouse models (76). The transcription factors liver X receptors (LXRβ and LXRα) are additional targets related to lipid metabolism in MDSC. As members of the nuclear hormone receptor family, LXRs play a role in activating the transcription of genes involved in cholesterol, fatty acid and glucose metabolism. LXR agonists have the potential to inhibit tumor growth and survival while inducing significant anti-tumor immune responses (108). In addition, the combination of RGX-104 with various immunotherapies, such as CAR-T and anti-PD-1 treatments, produced potent anti-tumor immune responses in mouse tumor models (109). A multi-center phase I dose-escalation trial of RGX-104, either alone or in combination with chemotherapy or immune checkpoint inhibitors, is currently ongoing in patients with lymphoma or metastatic solid tumors (NCT02922764).

Adenosine is an important metabolic and immune checkpoint regulator of tumor immunity (110). Several compounds targeting different components of the CD39-CD73-A2A/BR axis are currently in clinical trials as monotherapy or in combination with immunotherapies, with preliminary data suggesting good tolerability (111). In addition, these blocking agents can be combined with other therapies such as radiotherapy and chemotherapy to enhance their efficacy in cancer treatment (112, 113). Other potential strategies currently under investigation include co-inhibition of CD39 and CD73 (114, 115), dual inhibitors of A2AR and A2BR, and inhibition of both A2AR and CD73 (114).

In summary, in the context of cancer, the combination of strategies targeting MDSC metabolic activity with immunotherapeutic approaches holds promise for enhancing anti-tumor immunity.

## 7 Future prospects and challenges

MDSC are a major obstacle to effective immunity not only against tumors but also in other pathological conditions such as chronic infections. As in the case of cancer, targeting these cells may be a promising approach to enhance effective immunity and improve the treatment of these diseases. In the context of chronic infection, targeting MDSC metabolism offers several potential benefits. For example, by reducing the suppressive capacity of MDSC, the immune system can mount a more effective response against persistent pathogens, leading to improved clearance of infection and reduced disease burden. In addition, metabolic targeting may also enhance the efficacy of existing treatments, such as antibiotics and antiviral therapies, by creating a more favorable immune environment. However, there are challenges in implementing these strategies. Pathways are often shared by different cell types, and systemic inhibition could have unintended effects on other immune cells and tissues. It is therefore essential to develop targeted delivery systems and specific inhibitors that minimize off-target effects. For these reasons, therapeutic targeting of MDSC in chronic infections is currently less advanced than in cancer. While significant progress has been made in the development and clinical testing of therapeutic approaches targeting MDSC in the context of cancer, similar efforts in chronic infections are still in their infancy. The complexity of the interactions between MDSC and the immune system in the infection settings presents unique challenges. Strategies such as metabolic modulation, inhibition of pathways involved in MDSC expansion and function, and combination therapies are being investigated. However, the translation of these findings into effective clinical treatments for chronic infections requires further investigation and development.
